# Supplementary material for: A model for the dynamics of expanded CAG repeat alleles: ATXN2 and ATXN3 as prototypes
Source: Front Genet. 2023 Nov 14;14:1296614. doi: 10.3389/fgene.2023.1296614 (PMC10682950; doi:10.3389/fgene.2023.1296614)
Supplement: Supplementary file 1 [file DataSheet4.docx]

**Supplemental Material 4. General results of simulations performed with a new set of standard deviations of the fitness for the four allele categories.**

Table. Comparison between the general results obtained with two different assumed sets of values of SD of fitness of *ATXN3* alleles. Mean values of fitness were 1 for normal alleles and 1.5 for expanded alleles, according to Sena et al 2021b

| SD  of fitness | Lineages eliminated from  the population | Fixed alleles | | Lineages that remained in the population after  650 generations |
| --- | --- | --- | --- | --- |
|  |  | Lineages extinct after fixation | Lineages held as fixed |  |
| 0.25 for normal and expanded alleles * | 593 | 43 | 7 | 357 |
| 0.5 for expanded and 0.33 for normal alleles | 812 | 15 | 4 | 169 |

* Values assumed in the main text

Table. Comparison between the general results obtained with two different assumed sets of values of SD of fitness of *ATXN2* alleles. Mean values of fitness were 1 for normal alleles and 1.45 for expanded alleles, according to Sena et al 2021a

| SD of  fitness | Lineages eliminated from the population | Fixed alleles | | Lineages that remained in the population in the 650 generation |
| --- | --- | --- | --- | --- |
|  |  | Lineages extinct after fixation | Lineages held as fixed |  |
| 0.25 for normal and expanded alleles * | 933 | 67 | 0 | 0 |
| 0.5 for expanded and 0.33 for normal alleles | 994 | 6 | 0 | 0 |

* Values assumed in the main text
