# Supplementary material for: A model for the dynamics of expanded CAG repeat alleles: ATXN2 and ATXN3 as prototypes
Source: Front Genet. 2023 Nov 14;14:1296614. doi: 10.3389/fgene.2023.1296614 (PMC10682950; doi:10.3389/fgene.2023.1296614)

**Figure S1.** Fertility rates of women from the European general population in 2019 (data from European Statistical Office). (A) Fertility rate per age.  (B) Cumulative fertility rate.


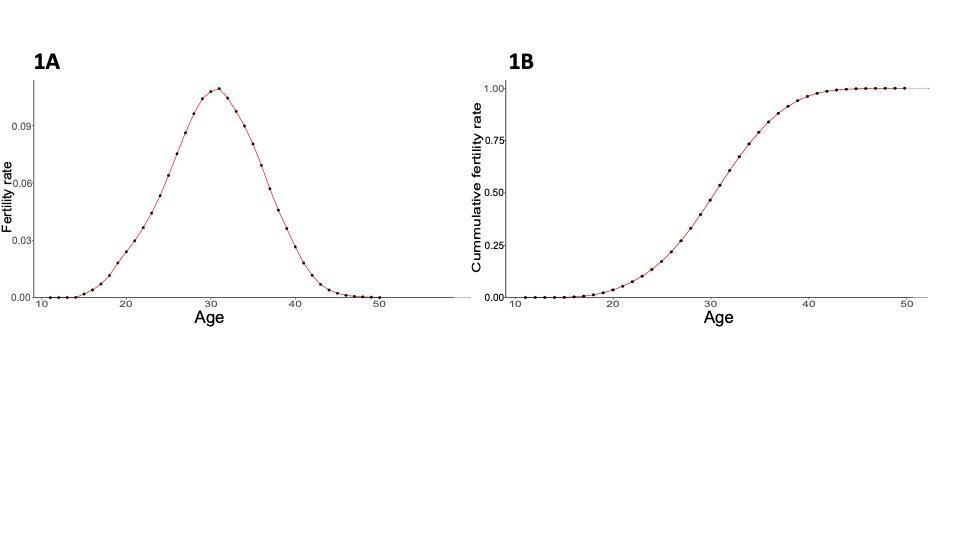


**Figure S2.** Fate of the 1,000 lineages simulated as descendants of one ancestral with a CAG expansion with an initial population frequency of 0.0001. (A) Frequency of the descendant alleles of the original CAG expansion in *ATXN2* founder, per generation  (B) Frequency of the descendant alleles of the original CAG expansion in *ATXN3* founder, per generation.


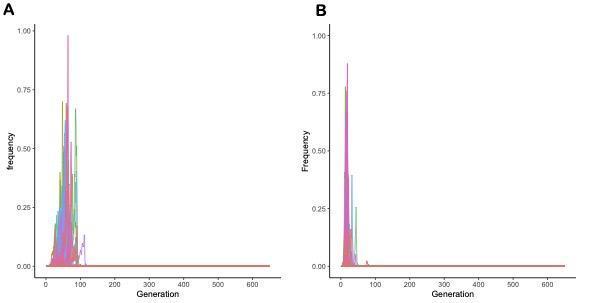


**Figure S3.** The median (IQR) number of generations in which expanded alleles remained present in *ATXN2* and *ATXN3*, in gene dropping simulations.


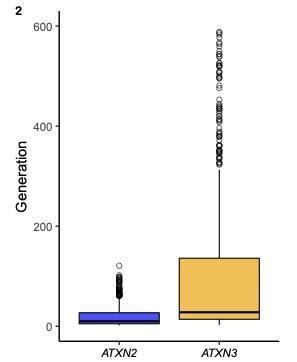

Supplement: Supplementary file 2 [file DataSheet3.docx]
