# Supplementary material for: A model for the dynamics of expanded CAG repeat alleles: ATXN2 and ATXN3 as prototypes
Source: Front Genet. 2023 Nov 14;14:1296614. doi: 10.3389/fgene.2023.1296614 (PMC10682950; doi:10.3389/fgene.2023.1296614)
Supplement: Supplementary file 3 [file DataSheet1.pdf]

Supplemental Material 1

The way the *antcoeff* was imputed in this paper, and the commands in R to perform the simulations.

The way the antcoeff was imputed in this paper

A) To calculate the area that each age contributes to the fertility area

trapz(area$age, area$"fertility_rate")

B) Transform the total area to a value of 1 and measure how much the reduction of 1 year of reproductive life reduces the total fertility rate

```
area$area_s = 0
for(i in 2: dim(area)[1]){
age_s = area$age[i]
aux = subset(area, age <= age_s)
area$area_s[i] = trapz(aux$age, aux$"2019.0")}
area$areapad = area$area_s / trapz(area$age, area$"2019.0")
```

Commands in R to perform the simulations

To perform frequency simulations in subsequent generations

```
p <- NULL
cag_rep <- NULL
for(j in 1:n_linhagens){
   p1 <-  initial frequency
   p2 <- NA
   cag_temp <- NA
   cag0 <- minor pathological cag
   print(paste0("lineage = ", n_linhagens))
   for(i in 1:generations){

      cag<- cag0+ if(cag <minor pathological cag) {rnorm(n = 1, mean instability of the unexpanded allele, standard deviation of unexpanded allele instability)} else {rnorm(n = 1,
expanded allele mean instability , standard deviation of expanded allele instability)}


       if(cag < minor pathological cag){
          w <- rnorm(n = 1, fitness not affected ,  fitness standard deviation unaffected)
          k <- rnorm(n = 1, non-expanded allele segregation distortion, standard deviation of unexpanded allele segregation)
       } else {
```

w <- rnorm(n = 1, ***fitness of those affected*** , fitness standard deviation unaffected)
k <- rnorm(n = 1,expanded allele segregation distortion , ***standard deviation of expanded allele segregation***)}

if(w <= 0) w <- 0

antcoeff <- coefant$coef[i]

ao <- ***decrease in AO by CAG**** cag + **(beta)**

```
  if(ao > 50){ antcoeff <- 1 }
  if(ao < 10){ antcoeff <- 0 }
  if(ao > 10 & ao < 50){ antcoeff <- predict(model, list(ao = ao), type="response") }

 cag0<-cag
  if(is.na(cag_temp)){
    cag_temp <- cag
  } else {
    cag_temp <- c(cag_temp, cag)
  }




    cag0<-cag
    if(is.na(cag_temp)){
      cag_temp <- cag
    } else {
      cag_temp <- c(cag_temp, cag)
    }
    if(antcoeff>1)antcoeff<-1
    print(paste0("generation ",  i, " - cag length = ", cag, " - ant. coeff = ", antcoeff))


    if(p1 != 0 & p1 != 1){
      if(is.na(p2)){
        p2 <- p1*w*antcoeff*2*k
      } else {
        p2 <- c(p2, p1*w*antcoeff*2*k)
      }
    } else {
      if(p1 == 0) p2 <- c(p2, 0)
      if(p1 == 1) p2 <- c(p2, 1)
    }
    if(p2[i] >= 1){ p2[i] <- 1 }
    if(p2[i] <= 0){ p2[i] <- 0 }
    p1 <- p2[i]

    w_tab[j, i] <- w
    k_tab[j, i] <- k
    ao_tab[j, i] <- ao
```

```
        antcoeff_tab[j, i] <- antcoeff
    }
    p[[j]] <- p2
    cag_rep[[j]] <- cag_temp
}
```
