# Supplementary material for: A model for the dynamics of expanded CAG repeat alleles: ATXN2 and ATXN3 as prototypes
Source: Front Genet. 2023 Nov 14;14:1296614. doi: 10.3389/fgene.2023.1296614 (PMC10682950; doi:10.3389/fgene.2023.1296614)

**Supplemental Material 2**

**The regression models that determined the expected reduction on AOfs per CAG repeat increases in the expanded tracts**

**SCA2 linear regression**

| Residuals | Min | 1Q | Median | 3Q | Max |
| --- | --- | --- | --- | --- | --- |
|  | -29.5021 | -5.8850 | -0.1614 | 4.7321 | 18.9874 |

| Coefficients: |  | Estimate | Std.Error | t value | Pr(>\|t\|) |
| --- | --- | --- | --- | --- | --- |
|  | (Intercept) | 110.1621 | 7.9035 | 13.938 | <2e-16 *** |
|  | CAG | -1.8724 | 0.1877 | -9.976 | 2.86e-16 *** |

Signif. codes: 0 ‘***’ 0.001 ‘**’ 0.01 ‘*’ 0.05 ‘.’ 0.1 ‘ ’ 1

Residual standard error: 8.299 on 91 degrees of freedom

Multiple R-squared: 0.5224, Adjusted R-squared: 0.5171

F-statistic: 99.52 on 1 and 91 DF, p-value: 2.864e-16


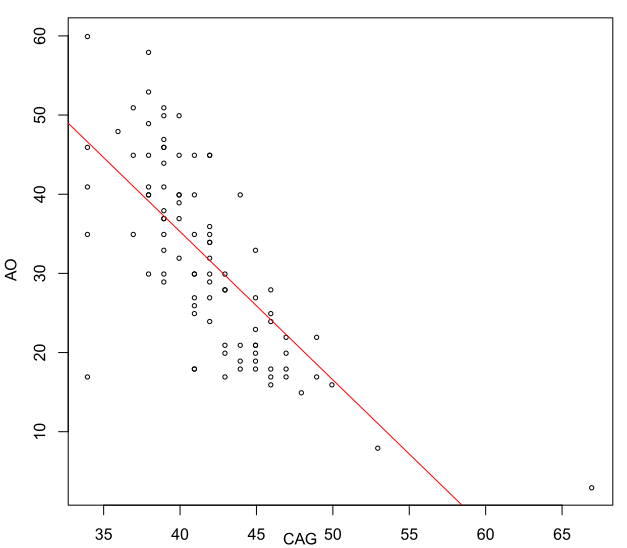


**SCA3 linear regression**

| Residuals | Min | 1Q | Median | 3Q | Max |
| --- | --- | --- | --- | --- | --- |
|  | -34.407 | -5.452 | 0.112 | 5.504 | 28.593 |

| Coefficients: |  | Estimate | Std.Error | t value | Pr(>\|t\|) |
| --- | --- | --- | --- | --- | --- |
|  | (Intercept) | 155.38853 | 3.69297 | 42.08 | <2e-16 *** |
|  | CAG | -1.65190 | 0.05182 | -31.88 | <2e-16 *** |

Signif. codes: 0 ‘***’ 0.001 ‘**’ 0.01 ‘*’ 0.05 ‘.’ 0.1 ‘ ’ 1

Residual standard error: 8.658 on 1111 degrees of freedom

Multiple R-squared: 0.4777, Adjusted R-squared: 0.4772

F-statistic: 1016 on 1 and 1111 DF, p-value: < 2.2e-16


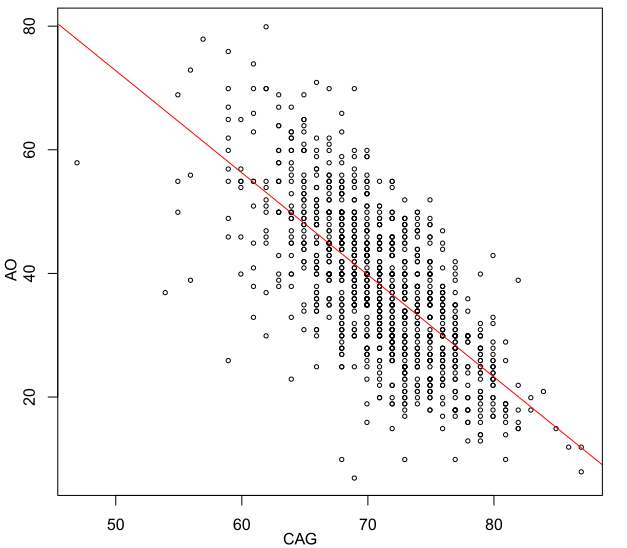

Supplement: Supplementary file 4 [file DataSheet2.docx]
